# Xenomelia: A Social Neuroscience View of Altered Bodily Self-Consciousness

**DOI:** 10.3389/fpsyg.2013.00204

**Published:** 2013-04-24

**Authors:** Peter Brugger, Bigna Lenggenhager, Melita J. Giummarra

**Affiliations:** ^1^Neuropsychology Unit, Department of Neurology, University Hospital ZurichZurich, Switzerland; ^2^Zurich Center for Integrative Human Physiology, University of ZurichZurich, Switzerland; ^3^Neuroscience Center Zurich, University of Zurich and ETH ZurichZurich, Switzerland; ^4^University Hospital of Child and Adolescent Psychiatry, University of BernBern, Switzerland; ^5^School of Psychology and Psychiatry, Monash UniversityClayton, VIC, Australia; ^6^Caulfield Pain Management & Research Centre, Caulfield HospitalCaulfield, VIC, Australia

**Keywords:** amputation, body integrity identity disorder, body modification, disability, psychiatry, neurology, sociology, medical ethics

## Abstract

Xenomelia, the “foreign limb syndrome,” is characterized by the non-acceptance of one or more of one’s own extremities and the resulting desire for elective limb amputation or paralysis. Formerly labeled “body integrity identity disorder” (BIID), the condition was originally considered a psychological or psychiatric disorder, but a brain-centered *Zeitgeist* and a rapidly growing interest in the neural underpinnings of bodily self-consciousness has shifted the focus toward dysfunctional central nervous system circuits. The present article outlays both mind-based and brain-based views highlighting their shortcomings. We propose that full insight into what should be conceived a “xenomelia spectrum disorder” will require interpretation of individual symptomatology in a social context. A proper social neuroscience of xenomelia respects the functional neuroanatomy of corporeal awareness, but also acknowledges the brain’s plasticity in response to an individual’s history, which is lived against a cultural background. This integrated view of xenomelia will promote the subfield of consciousness research concerned with the unity of body and self.

In times when an author can barely write about cognition without emphasizing its “embodied” aspects, it seems especially compelling to consider body-brain interactions in the field of consciousness studies or the cognitive neuroscience of “the self.” In fact, philosophers and scientists agree that knowledge about how the brain processes bodily sensations and plans executive action is key to the understanding of the experience of being a conscious self (Blanke and Metzinger, 2009). However, an individual’s bodily self-consciousness is not fully predefined by genes and neural circuits. It is constantly compared with others’ relationships to their bodies and evaluated against cultural norms about bodily appearance. In this article we will review work on xenomelia, one variant of the normal relationships between body and self, that is the desire of a healthy individual to have a fully functional limb amputated. We propose a *social neuroscience of xenomelia* that unifies neurological, psychological, and sociological approaches to bodily self-consciousness.

## Xenomelia: A Disorder of Bodily Self-Consciousness

While the term “xenomelia” is new (McGeoch et al., 2011), the condition is not. Several authors cite the eighteenth century case of a man who enforced the amputation of a leg from a surgeon at gunpoint (Johnston and Elliott, 2002; Hilti and Brugger, 2010). Later on, the psychiatric literature has described the desire for amputation as a paraphilia (Money et al., 1977; Everaerd, 1983) and around the turn of the century as an identity disorder focusing on one’s body configuration (“amputee identity disorder,” Furth and Smith, 2000; Smith, 2004). Most influential was the large-scale survey by First (2005), in which the term “body integrity identity disorder” (BIID) was coined. Fifty-two individuals were administered a structured telephone interview, which helped characterize the altered bodily self-consciousness that culminates in desire for amputation (Table 1). The findings established that (1) the condition is rooted in early childhood, (2) it is associated with marked distress, often leading to self-inflicted amputation attempts, (3) there is a male predominance, and recent studies show that women are more likely to desire bilateral amputations, and (4) it is, as a rule, accompanied by a socially non-conform attitude toward and admiration of “handicapped” individuals’, especially amputees’, bodies. These observations let First (2005, p. 919) define the disorder as “an unusual dysfunction in the development of one’s fundamental sense of anatomical (body) identity.” While the term BIID is most widely used in the current-day literature (First and Fisher, 2012), we prefer to use the label “xenomelia,” as it is purely descriptive and devoid of any interpretation. In particular, it neither suggests nor excludes the possibility that the desire for healthy limb amputation is in fact an identity disorder as defined in the DSM-IV. “Xenomelia” [from the Greek terms Xe
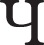

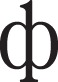
 (xeno) = foreign and μελoσ (melos) = limb] points to an estrangement of one or more of one’s limbs. It is important to emphasize the broad range in the wording sufferers use to capture this sense of estrangement. What follows are some typical firsthand descriptions extracted from the literature.

“*I can feel exactly the line where my leg should end and my stump should begin. Sometimes this line hurts or feels numb*.” (an individual with amputation desire; Blom et al., 2012, p. 2)

“*I feel myself complete without my left leg* … *I’m over-complete with it*” (individual with amputation desire; First, 2005, p. 922)

“*The soul feels as though it belongs to a body with only one leg. The body does not correspond to this inner reality*.” (man with amputation desire; Kasten, 2009, p. 17)

“*I feel the stump ends in my thighs and a strong ‘desire’ (I don’t have the right word for it) to live with two thigh stumps*.” (man with amputation desire; Kasten and Spithaler, 2009, p. 24)

“*I was eager for people to watch me, to see that my legs couldn’t move. [*…*] I was full of emotion. I felt whole for the first time in my life*.” (48-year-old woman with desire for paraplegia describing her feelings while pretending to be paralyzed and wheel-chair bound; Bruno, 1997; p. 247)

“*With BIID, the numbness goes beyond the legs. It seeps into my emotions*… *[*…*] I wandered in [to Transabled.org] through a link and I never left* … *it’s making the numbness feel not so shameful*.” (man with xenomelia; Davis, 2012; p. 611)

**Table 1:** **Six major questionnaire studies of xenomelia and selected findings**.

References (chronological order)	Type of survey	Sample size and characteristics^1,2^	Type of Xenomelia	Onset of the desire as reported retro-spectively^1^	Ratio legs to arms (%)	Ratio L to R to bilateral (%)	Specific findings emphasized by original author(s)
First (2005)	Telephone interview	*n* = 52 (four women, one intersex); mean age 48.6 (range 23–77); 61% het, 31% hom, 7% bi	Amputation desire	65% <age 8; 98% <16 (mean n.r.)	76:24^3^	55:27:18^4^	Learning (from Internet) that one’s desire is not unique provides tremendous relief; low prevalence of heterosexuality
Blanke et al. (2009)	Telephone interview	*n* = 20 (three women); mean age 48.4 (range 29–72); 95% het	Amputation desire	65% age 3–9 (mean 11.6)	80:20	35:20:45	Paraesthesia and hypoesthesia of affected body parts; high prevalence of migraine
Kasten (2009), Kasten and Spithaler (2009)	Standardized personality inventories	*n* = 9 men from early 30s to early 70s; 33.3% het, 55.6% hom, 11.1% bi	Amputation desire (*n* = 1 desire for paraplegia)	67% ≤age 8; mean 8, range 4–12	100:0	50:17:17 (1 n.r., 2 L/R alternating)	Preferred amputation site can vary over time; no clinically relevant elevations on OCD, psychoticism, neuroticism
Johnson et al. (2011)^5^	Internet questionnaire	*n* = 72 (eight women, three “other”), mean age 46 (SD = 16) 60% het, 25% hom, 8% bi	10% desire for paraplegia	n.r.	81:10 (rest arm-leg-combination)	42:28:30^6^	22% are non-right-handed; >33% indicate altered sensitivity on affected limb
Blom et al. (2012)	Standardized psychiatric inventories; Internet questionnaire	*n* = 54 (79.6% men); age range 18–76 ; 55.6% het, 27.8% hom, 16.7% bi	Desire for amputation (*n* = 30) and paraplegia (*n* = 24)	Mean 6.7 range 3–15	90:7^7^ (*n* = 1 with desire for tetra amelia)	37:30:33^8^	Low prevalence of heterosexuality; surgery reduced self- rated disability in a sub-sample (*n* = 7) of those with amputation desire
Giummarra et al. (2012)	Internet questionnaire	*N* = 16 (six women; age range 19–65 (median: 38.9 years)	Desire for paraplegia (*n* = 16)	Range: 4–16; mean and median = 9	100:0 (paralysis)	0:0:100	37.5% of cases were women, c.f. 4.4% of cases of amputation desire in previous samples. Sex differences may correspond to sex-related differences in cerebral lateralization.

*n.r., not reported; OCD, obsessive-compulsive disorder*.

*^1^Age in years*.

*^2^Het, heterosexual; hom, homosexual; bi, bisexual*.

*^3^100% = those 50 individuals wishing for a major limb amputation*.

*^4^100% = those 44 individuals, who specified laterality*.

*^5^Describes two surveys (total *n* = 97, but some individuals responded to both); numbers here refer to larger sample of survey 2*.

*^6^Over both surveys; 100% = all cases with amputation desire*.

*^7^In addition there were *n* = 2 with desire for blindness and *n* = 2 with other sorts of physical disability*.

*^8^100% = 30 individuals with limb amputation desire*.

The first few citations in the above list are mainly taken from authors propagating (or at least considering) a neurological, i.e., brain-based account of xenomelia. The focus here is altered sensation, or “paresthesias” (Blanke et al., 2009) localized to the undesired body part. More frequent are statements where the estrangement is less physical, but involves a vague (mind-based) feeling of non-belonging of the undesired limb(s) or the general notion of a disturbing “overcompleteness” of one’s body. Finally, xenomelic estrangement can transcend consciousness of one’s own body and manifest itself only in the social context, with the empathic perception or admiration of other peoples’ bodies. It is only by empathically comparing oneself to conspecifics, who are amputated, paralyzed, or otherwise “transabled” that one can anticipate possessing amputated limb(s) with stumps, prostheses, and mobility aids. As the last two quotes highlight, wholeness can be achieved by acting on one’s desire in public, or relief from numbness can be brought about by entering a virtual community of persons with supposedly similar suffering.

## Variants and Accompanying Features of Xenomelia

One frequent variant of the desire for amputation is the desire for paraplegia, i.e., the paralysis of both legs (Giummarra et al., 2012). From a neurological stance the peripheral absence of one or both legs and their paralysis due to spinal cord injury appear worlds apart. A person with the desire for paraplegia usually abhors the thought of amputation, while one with an amputation desire (even if bilateral) is typically convinced that being paralyzed from the waist down would not make him feel “in the right body.” However, with respect to some frequent correlates of xenomelia, the two variants are surprisingly similar (Blom et al., 2012 for a tabular comparison). The main phenomenological correlates of the different forms of xenomelia concern the erotic attraction to amputees or paraplegics and the urge to simulate the desired state.

Paraphilic pre-occupation with the desired body modification is reported by 46–87% of samples in published accounts of xenomelia (First, 2005; Blom et al., 2012; Giummarra et al., 2012) and preferably targets amputation or paralysis of the legs. While sexual arousal is rarely the primary explanation of the desire to change one’s own anatomy, it is worthy of attention with respect to the ontogeny and phenomenology of the condition. When considering functional neuroanatomy, the association of one’s consciousness for legs (compared to that for arms) and sexual feelings and behavior has its origins in the proximity of cortical regions representing legs and the sexual organs (Kell et al., 2005). It is well-documented that leg, but not arm (although, see Giummarra et al., 2011b) amputees experience referred genital sensations to their phantom limbs (Aglioti et al., 1994). In paraplegia, phantom leg sensations may also spread to the genital area, leading to full-blown orgasmic experiences (Avenarius and Gerstenbrand, 1967). Many paraphilias can be traced to discrete events or experiences during childhood, when children are enthusiastic imitators through the process of developing their self-consciousness, often engaging in socially inappropriate mimicry and role-play (Whiten et al., 2009). In xenomelia, as with other paraphilias, such discrete experiences may become the fetishistic targets during puberty, likely by conditioning (Koksal et al., 2004), into the individual’s sexual interests and masturbatory fantasies (Abel et al., 2008). Rather than forming a springboard to psychodynamic elaborations, the lower limb predominance together with the erotic components of xenomelia may illustrate that “body schema” involves more than proprioceptive, somatosensory, and motor aspects of an individual’s development, but is also tightly linked to a person’s sexual identity (Schilder, 1935; Money, 1984; De Preester, 2011). It may even transcend individual consciousness and embrace cultural dimensions. Thus xenomelia and fetishistic foot-binding practices in traditional Chinese culture probably rest on similar neural mechanisms (McGeoch, 2007).

The second sign frequently accompanying xenomelia is the simulation of the desired state. Persons with xenomelia typically spend a great deal of time obsessing about their desired or “beloved” body-state (Sorene et al., 2006; Kasten, 2009). The majority report “pretending” behaviors, whether these simply involve mental imagery, or motor action in accordance with their desired body form (e.g., binding the legs, sitting on them, using tourniquets to reduce lower limb perception, transferring to/from a wheel chair without using the legs, or trying not to engage the undesired limbs with motor tasks; Riordan and Appleby, 1994; First, 2005; Giummarra et al., 2012). Individuals at the more “extreme” end of the spectrum of xenomelia meet at least some of the criteria for obsessive-compulsive disorder. While Oddo et al. (2009) found some support for OCD in people with xenomelia, the obsessions tend to be limited to their altered bodily self-consciousness.

Given that both paraphilic and pretending components are equally characteristic to the amputation and paraplegia variant of xenomelia we propose that both variants “belong to the spectrum of BIID” (Giummarra et al., 2012, p. 35). The desire to become incontinent, castrated (Roberts et al., 2008), deaf (Veale, 2006), blind (Johnson et al., 2011), or suffering from neurological dysfunction (Kolla and Zucker, 2009) may be part of this spectrum.

## Xenomelia: Mind-Based vs. Brain-Based Interpretations

**Figure 1:**
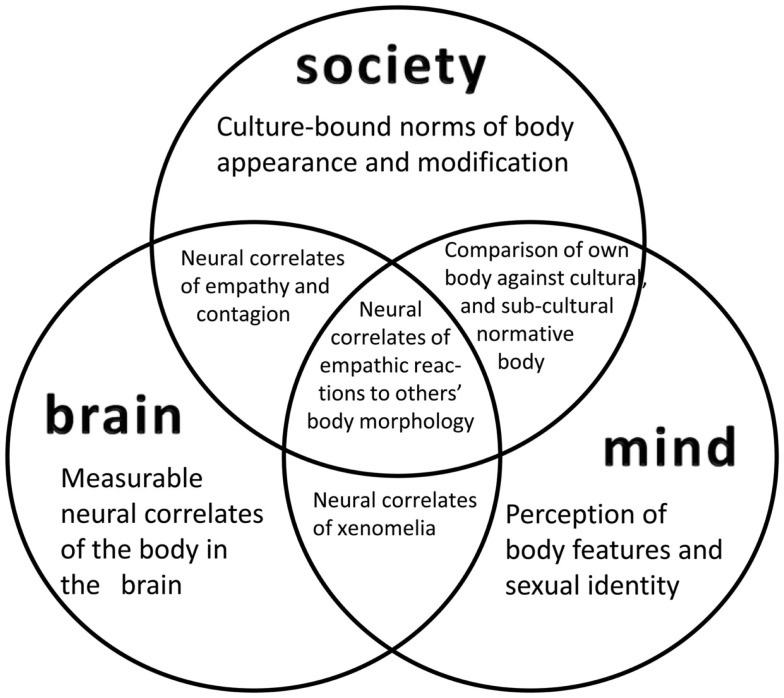
**A view of xenomelia research that integrates three approaches**. While the crosstalk between neurological (“brain-based”) and psychological (“mind-based”) approaches is commonplace, anthropological, and social studies of bodily appearance and its modifications have been neglected in neuroscience accounts. A social neuroscience view of xenomelia respects the interactions between an individual’s perception of the own body in relation to others’ bodies and as influenced by normative standards. It will also explore the neural correlates of these interactions and investigate, for instance, the constraints of empathy by social norms, or the impact of a person’s preconceptions of a “handicapped body” on brain function and structure.

As a rare and peculiar state of self-consciousness, xenomelia has been approached from both psychiatric and neurological fields. We designate the former approaches as “mind-based,” even if their conclusions might be formulated in neuro-terms. Mind-based approaches are indispensable to the understanding of any neuropsychiatric conditions as, even if we adhere to a brain-mind identity view of consciousness, mind and brain *are* two different things (Figure 1). The language appropriate for speaking about minds is not appropriate for speaking about brains (Horne, 1994). Just as there is no “suffering brain,” there is no “atrophic mind.” The many case reports on xenomelia and the systematic group studies that historically followed them (Table 1) are essentially mind-based. They provide important clues to development and phenomenology of the condition, to (purportedly) eliciting events and individual strategies of coping. As can be seen from the specific findings highlighted by the original authors, the six major questionnaire studies have painted a multicolored picture of xenomelia. The convergence across studies paves the way for prospective neurologically oriented investigations, particularly focusing on the neural correlates of the primacy of legs over arms, left-sided symptom preponderance (Table 1) and the often precise demarcation line between accepted and non-accepted body territories.

Brain-based accounts of xenomelia were originally motivated by comparing xenomelic individuals’ bodily self-consciousness with that reported by neurological patients after brain damage. Syndromes like (hemi)asomatognosia (felt absence of body parts; Dieguez et al., 2007), somatoparaphrenia (lack of limb ownership; Vallar and Ronchi, 2009), or misoplegia (hatred toward a body part; Loetscher et al., 2006) are most often mentioned. They are indicative of right parietal cortex dysfunction. Direct evidence for impaired parietal lobe functions in four subjects with xenomelia comes from McGeoch et al.’s (2011) magnetoencephalographic study, which showed an unresponsive superior parietal lobule to tactile stimulation of specifically “undesired” parts of the body. Hilti et al. (2013) described structural abnormalities in this structure and the right anterior insula of 13 men with xenomelia, supporting parallels between xenomelia and disturbed self-consciousness in disorders like somatoparaphrenia (Karnath and Baier, 2010). Despite the fact that the insula has advanced, in recent years, to an island representing pretty much every thinkable function (Craig, 2011 for overview), we consider the insular anomalies to be meaningful: not only is the right insular cortex known to be key for integrating interoceptive bodily feelings, but it is a core region for the convergence of somesthesis and sexual arousal (Hilti et al., 2013). We predict that we will see an increased awareness for the neurological underpinnings of xenomelia in the near future. Nevertheless, the clear shortcomings of a purely brain-based approach should not be overlooked (Sedda, 2011); among many other clinical observations, the switching of a longstanding desire from a left-sided to a right-sided leg amputation (Kasten and Stirn, 2009) or the sudden occurrence of new amputation desires after previous ones were satisfied (Sorene et al., 2006) are not easily explainable by the sole reference to neural mechanisms. Furthermore, any observed correlations between mind and matter is often silently interpreted in a unidirectional way, i.e., from brain to mind. Brang et al. (2008, p. 1306) considered that the study of xenomelia “may provide key insights into the question of how neural activity gives rise to mental phenomena” (p. 1306). From this view point, they interpret their finding of differential skin response to pain in affected vs. non-affected body parts in two xenomelia patients as evidence for a congenital brain dysfunction. Interpretations like this imply that states of the mind must ultimately be *caused* by states of the brain. This view is not in line with the focus of the present *Research Topic* nor is it necessarily supported by the current literature on neuroplasticity. While interest in brain plasticity was originally triggered from observing behavioral consequences of cortical reorganization after deafferentation, the focus has now shifted toward “experience-dependent neuroplasticity.” Such plasticity extends far beyond the juvenile period (Lillard and Erisir, 2011), and comprises sensorimotor training (Bezzola et al., 2012) and musical experience (Parbery-Clark et al., 2012) in the aging. Together with evidence for epigenetic alterations in both mind and neural matter (Ventura-Junca and Herrera, 2012), and yet other socio-cultural factors (see below), such communication between nature and nurture should not be hastily interpreted as a primacy of either brain or mind. Thus, the correlation between structural cortex alterations and the strength of an individual’s amputation desire (Hilti et al., 2013) could indicate that xenomelia is the consequence of early, perhaps even prenatal neural development (Hilti and Brugger, 2010). However, equally plausible is the assumption that years, if not decades, of a hostile attitude directed to a part of the body, and potential behavior-induced peripheral atrophy (e.g., see Bensler and Paauw, 2003; Storm and Weiss, 2003), may have produced cortical changes.

## Xenomelia Spectrum Disorders: Integrating Brain, Mind, and Society

Brain-based and mind-based approaches, even in their unification, will not solve the riddle of xenomelia. Bodily self-consciousness is continuously shaped by culture-bound norms regarding body appearance and the tolerated extent to which this appearance may be modified (Jordan, 2004). A full understanding of non-psychotic individuals’ feeling of “being in the wrong body” and their desire to correct the mismatch between body and self by massive modifications of the prototypical, four-limbed corporeal morphology can only be reached by respecting the crosstalk between brain, mind, and society (Figure 1; notably also applying to related alterations of bodily self-consciousness; see Giummarra et al., 2011a, for review).

The social dimension of body-image, as unpopular as it may be in current-day neuroscience, has been fully appreciated in former times. When writing on “the sociology of the body-image,” Schilder (1935, part III) highlighted that “There exists a deep community between one’s own body-image and the body-image of others. In the construction of the body-image there is a continual testing to discover what could be incorporated in the body […] the body-image is a social phenomenon” (p. 217). The continual comparison between the self and others is at the heart of empathic reactions during encounters with those who live in different bodies. Almost half of xenomelic persons consider such encounters highly meaningful life events, for some they are even causative triggers of their later desires (Aoyama et al., 2012). It is possible that one prerequisite of xenomelia is an overemphatic response and an exaggerated expression of mimicry, i.e., the imitative incorporation of another person’s postural and gestural displays (Chartrand and Bargh, 1999; van Baaren et al., 2009). This incorporation may lead to identification with a body-image that does not correspond with one’s own anatomy and functionality. Although the empathic merging of one’s own with others’ bodies is greatly facilitated by vision and the cutaneous senses (Morrison et al., 2010) it does not necessarily rely on real-life encounters with other people. Language is a powerful mediator of the construction of intersubjective representations (Merleau-Ponty, 1945/1962), and recent research has focused on the particular role of internet communication. “Prosumption” is the blurring of production and consumption in the communicative (originally economic; Toffler, 1980) context. While we all prosume as communicating members of a society, cyberspace-mediated prosumption is especially prominent in shaping the identity of marginalized or stigmatized individuals. Davis (2012) analyzed the information exchange among members of an internet site devoted to persons with xenomelia. She found that transableism has become a fluid identity construct that is shaped by, and at the same time shapes, the identity of fellow bloggers’. Social media has likewise been identified as having a powerful impact on the creation of “transient mental illnesses” (Hacking, 1999; Baubet et al., 2007) or “contagious desires” (Elliott, 2000). While it is debatable whether xenomelia is an illness of the mind, brain, or a culture-bound syndrome, its inclusion in DSM-V would undoubtedly present a Janusian face to the “transabled” community. On the one hand it might pave the way for legally sanctioned surgery in response to intolerable distress (Ryan, 2009), on the other hand it would contribute to further marginalize a form of bodily self-consciousness that may be viewed as a “non-normative form of embodiment” (Sullivan, in press) rather than an illness (a similar issue is central to disorders of gender identity and sexual development; Reis, 2007; Lawrence, 2009, 2010).

A future social neuroscience of bodily self-consciousness will acknowledge that, as mighty as social processes like prosumption might be, they do not invalidate neurological findings that differentiate people with xenomelia from people without. Empathic resonance can change basic perceptual processes (Lopez et al., 2013) and corresponding neural changes are to be expected. The integration of social, psychological, and neurological views of xenomelia will offer a unique way to explore the reciprocal influences between brain, mind, and society in relation to corporeal awareness and the experience of the self.

## Conflict of Interest Statement

The authors declare that the research was conducted in the absence of any commercial or financial relationships that could be construed as a potential conflict of interest.
